# Prenatal exposure to polycyclic aromatic hydrocarbons and cognitive dysfunction in children

**DOI:** 10.1007/s11356-014-3627-8

**Published:** 2014-09-26

**Authors:** Wiesław A. Jedrychowski, Frederica P. Perera, David Camann, John Spengler, Maria Butscher, Elzbieta Mroz, Renata Majewska, Elżbieta Flak, Ryszard Jacek, Agata Sowa

**Affiliations:** 1 Chair of Epidemiology and Preventive Medicine, Jagiellonian University Medical College, 7, Kopernika Street, Krakow, Poland; 2 Columbia Center for Children’s Environmental Health, Mailman School Public Health, Columbia University, New York, NY 10027 USA; 3 Department of Analytical and Environmental Chemistry, Southwest Research Institute, San Antonio, TX 78238 USA; 4 Harvard School of Public Health, Harvard University, Boston, MA 02138 USA; 5 Polish-American Institute of Pediatrics, Jagiellonian University Medical College, Krakow, Poland

**Keywords:** Epidemiologic study, Children, Cognitive function, Prenatal PAH exposure

## Abstract

Polycyclic aromatic hydrocarbons (PAHs) are widespread environmental pollutants produced by combustion of fossil fuel and other organic materials. Both experimental animal and human studies have reported the harmful impacts of PAH compounds on fetal growth and neurodevelopment, including verbal IQ of children. Here, we have assessed the association between cognitive function of children and prenatal PAH exposures. The study is part of an ongoing, longitudinal investigation of the health effects of prenatal exposure to air pollution on infants and children in Krakow, Poland. The subjects in this report included 170 children whose mothers were enrolled to the study in the first or second trimester of pregnancy whose cord blood were tested for PAH–DNA adducts and who were assessed at age 7 using the Wechsler Intelligence Scale for Children-Revised (WISC-R). The outcome of a priori interest was depressed verbal IQ index (DepVIQ), which is the difference between WISC-R performance and verbal IQ scores. Prenatal PAH exposure was measured by cord blood PAH–DNA adducts, an individual dosimeter, integrating exposure from various sources of exposure over the gestational period. The estimated effect of prenatal PAH exposure on cognitive function was adjusted in multivariable regression for a set of potential confounders (child’s gender, parity, maternal education, breastfeeding practice, environmental tobacco smoke (ETS), and postnatal PAH exposure). The prevalence of DepVIQ was significantly higher in children with detectable PAH–DNA adducts compared to those with undetectable adducts (13.7 vs. 4.4 %,). Binary multivariable regression documented that the relative risk of DepVIQ increased threefold with a ln-unit increase in cord blood adducts (relative risk (RR) = 3.0, 95 % confidence interval (CI) 1.3–6.8). Postnatal PAH exposure also increased the risk of DepVIQ (RR = 1.6, 95 % CI 1.1–2.5). Long-term exclusive breastfeeding (at least 6 months) showed a protective effect (RR = 0.3, 95 % CI 0.1–0.9). In conclusion, these results provide further evidence that PAHs are harmful to the developing fetal brain with effects extending through childhood, with implications for the academic success of the children.

## Introduction

Early cognitive development is vital for an individual’s ability to learn, adjust, and take advantage of the opportunities available in various environments (Deary 2010; Hunter 1986; Moffitt et al. 1981). It has been demonstrated that individuals scoring higher on intelligence tests in early childhood are more likely to achieve higher education levels and socioeconomic status and have greater success in professional careers (McCall 1977). Attention to the impact on children’s cognitive dysfunction of ambient air pollutants such as tobacco smoke (Bauman et al. 1991; Eskenazi and Castorina 1999; Johnson et al. 1999; Weitzman et al. 2002; Yolton et al. 2005), diesel exhaust pollutants (Wang et al. 2009), or exposure to polycyclic aromatic hydrocarbons (PAHs) (Perera et al. 2011) has recently increased (Brown et al. 2005), and our previous study in the present cohort reported that prenatal PAH was associated with lower intelligence scores at age 5 (Edwards et al. 2010).

PAHs belong to a group of chemical compounds formed during the incomplete combustion of organic material; the best known member of this class of compounds is benzo[a]pyrene (BaP). The PAH compounds are ubiquitous and have been found in polluted air in occupational and urban environments, tobacco smoke, and broiled foods (IARC 2010; Lijinsky 1991; Phillips 1999; US EPA 2005; WHO 2001). They readily cross the placenta (Castano-Vinyals et al. 2004; Godschalk et al. 2003; Kihlstrom 1986; Schulte and Perera 1993;); however, the estimated transplacental dose of PAH is about ten times lower than the dose to maternal tissues (Perera et al. 2005). Transplacental exposure to PAH leads to the formation of DNA adducts in the fetus (Neubert and Tapken 1988; Srivastava et al. 1986), which are considered a molecular dosimeter of PAH absorbed over the prenatal period. PAH–DNA adducts reflect individual differences in exposure, absorption, and distribution of the chemicals, metabolism to DNA reactive forms, and detoxification to less reactive intermediates as well as repair of DNA damage (Schocket 1999; Shuker 2002).

In our study, the Wechsler Intelligence Scale for Children-Revised (WISC-R), which is a valid and reliable measure of general intelligence in children and the most widely used instrument for the assessment of child intelligence (Wechsler 1991, 1994, 2004), was applied to assess the verbal IQ (VIQ) and performance IQ (PIQ) of 7-year-olds. Cognitive dysfunction in children was measured by the depressed verbal IQ index (DepVIQ), defined as a difference between PIQ and VIQ scores corresponding the 90th percentile. As DepVIQ was found to be associated with poor academic achievement in children, it has lead to increased interest in the use of DepVIQ as a marker for possible academic difficulties in children (Moffitt and Silva 1987).

The main purpose of this analysis was to assess the association between depressed verbal IQ score and prenatal PAH exposure measured by cord blood PAH–DNA adducts. The effect of prenatal PAH exposure was adjusted for a set of potential confounders such as maternal education, gender of child, parity, gestational age, breastfeeding practice, environmental tobacco smoke (ETS), and postnatal exposure to indoor PAH.

## Materials and methods

This study is part of an ongoing, longitudinal investigation of the health effects of prenatal exposure to air pollution on infants and children in Krakow, Poland. As described previously (Jedrychowski et al. 2003), between November 2000 and March 2003, we initially recruited women between 8 and 13 weeks of pregnancy who registered at prenatal health care clinics in Krakow inner city area, where they had also lived for at least a year preceding screening. Eligibility criteria included the following: ≥18 years of age, women, nonsmoking, singleton pregnancies, no current occupational exposure to PAH or any other known developmental toxicants, no history of illicit drug use, pregnancy-related diabetes, or hypertension. Five hundred five were fully enrolled (met the eligibility criteria, had prenatal PAH monitoring data, and provided cord blood samples). Out of all fully enrolled women, 484 delivered term babies (>36 weeks of gestation). This report concerns the 170 children who had data on household airborne PAH monitoring at the age 3 and valid psychological WISC-R testing performed at the age 7. Informed consent was obtained from all subjects. The study was approved by the ethics committee of Jagiellonian University and the Institutional Review Board of the New York Presbyterian Medical Center.

Detailed data on maternal education was used as a proxy for social class, intellectual ability, and quality of parenting. Breastfeeding initiation and its duration were based on the answers from interviews taken at regular 3-month intervals over the postpartum period. Mothers were asked whether the infant had ever been breastfed and, if so, the age of the baby (in months) when exclusive breastfeeding was stopped. Exclusive breastfeeding was assumed if the child received only breast milk and no other liquids or solids with the exception of medicine or mineral supplements. Data on the number of cigarettes smoked daily by all household members was used to assess environmental tobacco smoke (ETS) at home during the prenatal and postnatal periods.

### Dosimetry of PAH–DNA adducts

Samples of umbilical cord blood (30–35 mL) collected at delivery were transported to the laboratory immediately after collection. The buffy coat, packed red blood cells, and plasma were separated and stored at −70 °C. BaP–DNA adducts in extracted WBC DNA white blood cells were analyzed using the HPLC-fluorescence method of Alexandrov et al. (1992), which detects BaP tetraols, as previously described (Perera et al. 2005). The assay gives zero values when unexposed calf thymus DNA is tested (D. Tang, personal communication). The method has a coefficient of variation of 12 % and a lower limit of detection of 0.250 adducts per 10^8^ nucleotides. Samples below the limit of detection were assigned a value of 0.125 per 10^8^.

### Indoor airborne PAH monitoring

Study participants were monitored for exposure to airborne PAH postnatally at the age 3 using personal environmental monitoring sampler (PEMS) developed at the Harvard School of Public Health (Dr J. Spengler). The sampler was located in the main part of the household during a consecutive 48-h period. The sampling pump draws air through a polyurethane sampler (PUF) to measure PAH. After sampling, the field samplers were frozen and shipped on dry ice to the Southwest Research Institute in Texas. Personal air monitoring data was given a quality assurance (QA) score (0–3) for flow rate, flow time, and completeness of documentation. A final QA score of 0 (highest quality) or 1 (high quality) was required for inclusion. Air samples were analyzed at the Southwest Research Institute for levels of pyrene and eight carcinogeneic PAH. Determination of total PAH concentration (benzo(a)anthracene, benzo(b) fluoranthene, benzo(k)fluoranthene, benzo(g,h,i) perylene, benzo(a)pyrene, chrysene/isochrysene, dibenzo(a,h)anthracene, indeno(1,2,3-c,d)pyrene, and pyrene) in extracts was performed. Chemical procedures in the analysis of the collected samples were described elsewhere (US EPA 1999). In the present analysis, only total PAH measurements were considered.

### Mental development testing of children

At age 7, the WISC-R was administered by trained researchers. It has been found to be a good measure of both inductive and deductive reasoning, and it also measures knowledge and skills primarily influenced by biological and socio-cultural factors. The WISC-R includes questions of general knowledge, traditional arithmetic problems, vocabulary, completion of mazes, and arrangements of blocks and pictures and yields three IQ (intelligence quotient) scores, based on an average of 100 as well as subtests and index scores. WISC-R subtests measure specific verbal and performance abilities. The child’s VIQ is derived from scores on six of the subtests: information, digit span, vocabulary, arithmetic, comprehension, and similarities. The child’s PIQ, a measure of nonverbal intellectual abilities, is derived from scores on seven subtests: picture completion, picture arrangement, block design, object assembly, coding, mazes, and symbol search.

Verbal and performance IQs were rated using the method recommended in the manual (Wechsler 1994). The difference between values of the PIQ and VIQ is called a discrepancy score; a positive PIQ-VIQ means PIQ is greater than VIQ (DepVIQ); and a negative score indicates the reverse pattern. A statistical abnormality is defined to include PIQ–VIQ discrepancy scores equal to or greater than the 90th percentile in absolute value. In our sample, the criterion yielded cutoff scores of 22 IQ points. These scores at the 90th percentile rank are almost identical to those provided by Kaufman (1980).

The Wechsler scales were standardized for Polish children and are meant to be representative of the Polish population. The practical standardization of these tests was done during team practice sessions with Ms. Maria Butscher, a psychologist from the Jagiellonian University Medical College, who subsequently evaluated the IQ scoring.

### Maternal intelligence testing

As maternal intelligence is a known correlate of child cognitive development, we administered the Test of Nonverbal Intelligence (TONI-3) to the mothers at the 4th year of follow-up. The TONI-3 is a language-free measure of general intelligence, considered to be relatively free of cultural bias (DeMauro 2001).

### Statistical data analysis

In the initial part of the analysis, the distribution of various parameters related to the children under study was described. Chi-square statistics (nominal variables) and analysis of variance (numerical variables) tested differences between subgroups of children with depressed and nondepressed VIQ scores. Following descriptive univariate analysis, we used the multivariable binary regression GLM model to explore the relationship between DepVIQ as a binary variable using the cutoff of 22 points (≥90th percentile) and the level of cord blood PAH–DNA adducts (ln-transformed) adjusted for a set of a priori selected covariates (postnatal indoor PAH exposure, maternal education, child’s gender, parity, breastfeeding practice, and prenatal and postnatal ETS). As the correlation coefficients between cognitive scores achieved by children and maternal education (number of schooling years) and maternal IQ assessed by TONI test did not differ, we have chosen to consider only maternal education as a proxy for maternal intellectual ability and quality of parental care. Statistical analysis was performed by the statistical software STATA version 12.1 and two-sided *p* < 0.05 was considered statistically significant.

## Results

General characteristics of the sample are presented in Table 1. The characteristics of the subjects included in the analysis did not reveal significant differences compared with the group of children who were not included due to missing data (Table 2) indicating that the sample included in the analysis was representative of the overall study population.

**Table 1:** Characteristics of the study sample overall and according to the level of cord blood PAH–DNA adducts

Variables			Total *N* = 170	Cord blood PAH–DNA adducts		*p* for difference between adduct groups
				Detectable	Nondetectable	
				*N* = 102	*N* = 68	
Maternal age		Mean	27.64	27.27	28.21	0.0934
		SD	3.583	3.704	3.339	
Maternal education (years of schooling)		Mean	15.55	15.38	15.81	0.3373
		SD	2.831	2.740	2.964	
Parity	1	*n* (%)	118 (69.4)	73 (71.6)	45 (66.2)	0.5635
	≥2	*n* (%)	52 (30.6)	29 (28.4)	23 (33.8)	
Gender	Boys	*n* (%)	80 (47.1)	44 (43.1)	36 (52.9)	0.2723
	Girls	*n* (%)	90 (52.9)	58 (56.9)	32 (47.1)	
Gestational age (weeks) >36		Mean	39.61	39.56	39.69	0.4800
		SD	1.193	1.199	1.188	
Birth weight (g)		Mean	3445.6	3418.8	3485.9	0.3498
		SD	456.67	458.57	454.20	
Length at birth (cm)		Mean	54.93	54.84	55.06	0.6068
		SD	2.666	2.775	2.509	
Head circumference (cm)		Mean	33.97	33.93	34.02	0.7215
		SD	1.487	1.517	1.451	
Breastfeeding exclusive >6 months		*n* (%)	43 (25.3)	29 (28.4)	14 (20.6)	0.3308
Prenatal ETS		*n* (%)	44 (25.9)	28 (27.5)	16 (23.5)	0.6942
Postnatal PAH (indoor)		Mean	47.01	43.86	51.73	0.4351
		SD	64.19	66.72	60.37	
Postnatal ETS (1–7 age)		*n* (%)	41 (25.6)	25 (26.3)	16 (24.6)	0.9541
		Missing data	10	7	3	
WISC-R IQ at age 7:						
IQ verbal scale		Mean	119.4	119.3	119.4	0.9530
		SD	11.64	12.32	10.62	
IQ nonverbal scale		Mean	124.1	124.8	123.0	0.3930
		SD	13.74	13.45	14.18	
IQ full scale		Mean	123.9	124.3	123.4	0.6312
		SD	11.76	11.90	11.61	
Depressed verbal IQ (PIV-VIQ) ≥22		*n* (%)	17 (10.0)	14 (13.7)	3 (4.4)	0.0850

**Table 2:** Comparative characteristics of the children who took part in the follow-up and those who dropped from the study

Variables			Total	Included	Not included	*p* for difference
			*N* = 484	*N* = 170	*N* = 314	
Maternal age		Mean	27.55	27.64	27.50	0.6792
		SD	3.580	3.583	3.583	
Maternal education (years of schooling)		Mean	15.56	15.55	15.57	0.9481
		SD	2.759	2.831	2.724	
Parity	1	*n* (%)	307 (63.4)	118 (69.4)	189 (60.2)	0.0559
	≥2	*n* (%)	177 (36.6)	52 (30.6)	125 (39.8)	
Gender	Boys	*n* (%)	248 (51.2)	80 (47.1)	168 (53.5)	0.2081
	Girls	*n* (%)	236 (48.8)	90 (52.9)	146 (46.5)	
Gestational age (weeks) >36		Mean	39.54	39.61	39.50	0.2903
		SD	1.141	1.193	1.111	
Birth weight (g)		Mean	3443.0	3445.6	3441.6	0.9229
		SD	435.90	456.67	424.97	
Length at birth (cm)		Mean	54.75	54.93	54.65	0.2672
		SD	2.615	2.666	2.586	
Head circumference (cm)		Mean	33.91	33.97	33.88	0.5027
		SD	1.391	1.487	1.338	
Breastfeeding exclusive >6 months		*n* (%)	133 (27.5)	43 (25.3)	90 (28.7)	0.4929
Prenatal PAH		Mean	52.02	43.00	56.88	0.0375
		SD	66.37	55.34	71.23	
		Missing data	50	18	32	
Prenatal ETS		*n* (%)	130 (26.9)	44 (25.9)	86 (27.4)	0.8030
Postnatal PAH (indoor)		Mean	44.17	47.01	40.59	0.3430
		SD	58.62	64.19	50.77	
		Missing data	179	0	179	
Postnatal ETS (1–7 age)		*n* (%)	67 (24.7)	41 (25.6)	26 (26.1)	0.7872
		Missing data	213	10	203	

**Fig. 1:**
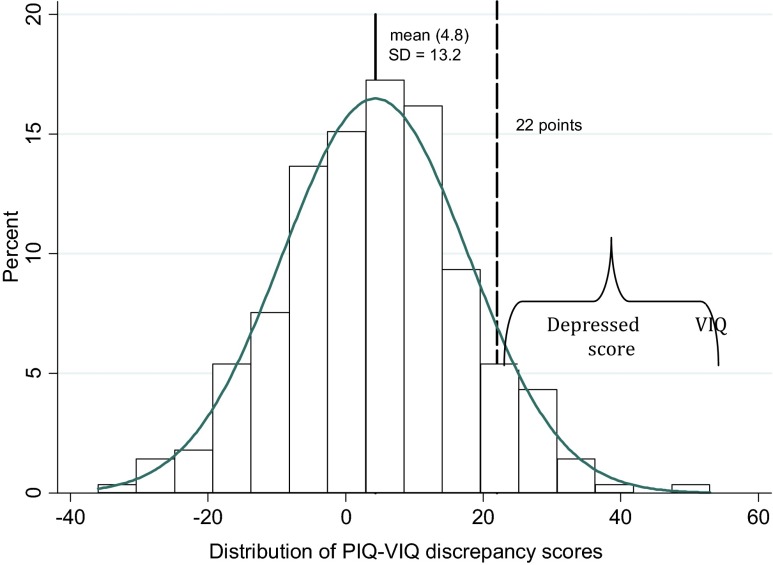
Distribution of PIQ-VIQ discrepancy scores in the study sample

The geometric mean value of the cord blood PAH–DNA adducts was 0.23 per 10^8^ nucleoides (95 % CI 0.21–0.25 per 10^8^ nucleoides). In the study sample, 60 % of newborn children had detectable cord blood PAH–DNA adducts with a median adduct level of 0.33 adducts per 10^8^ nucleotides. The overall mean VIQ score in the study population was lower (mean = 119.6; 95 % confidence interval (CI) 117.9–121.4) than the PIQ score (mean = 124.3; 95 % CI 122.2–126.4), but the difference was statistically insignificant. The mean discrepancy score between PIQ and VIQ was 4.7 points (95 % CI 3.0–6.5), and its distribution was perfectly normal (Fig. 1).

**Fig. 2:**
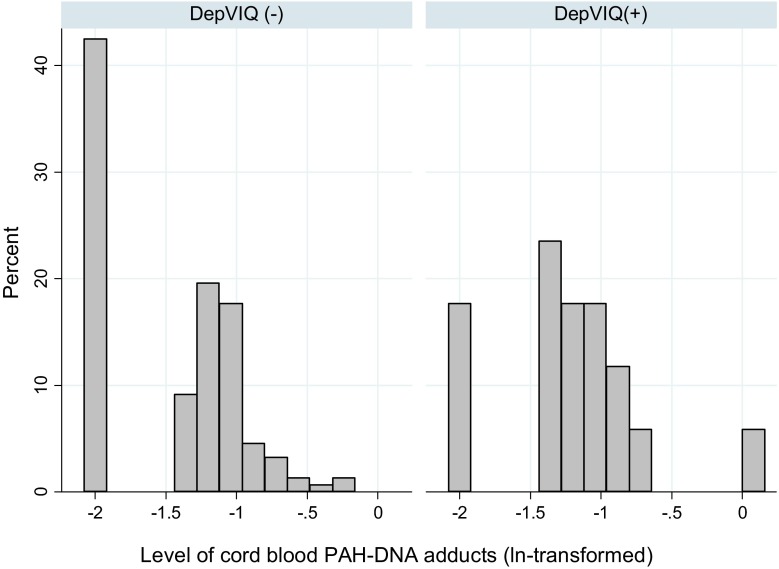
Cord blood adducts’ levels grouped by DepVIQ category

The children with depressed verbal IQ score (DepVIQ) of at least 22 points had significantly lower VIQ by 10.5 points and higher PIQ by 18.2 points than those in the control group of children (nondepressed VIQ) (Table 3). The distribution of cord blood adducts was shifted to the right in the group of children with DepVIQ (Fig. 2), and the prevalence of DepVIQ was significantly higher in children with detectable PAH–DNA adducts compared to those with undetectable adducts (13.7 vs. 4.4 %, chi-square = 4.245, *p* = 0.039). However, DepVIQ was found to be less prevalent in children exclusively breastfed for the 6-month period or longer than in those who were breastfed for a shorter time (6.3 vs.14.9 %, chi-square = 3.446, *p* = 0.063). Child’s gender, maternal education (years of schooling), and parity were not associated with DepVIQ.

**Table 3:** Wechsler IQ scores in children with depressed verbal IQ score and in the control group

Variables	Controls *N* = 153		Depressed verbal IQ score *N* = 17		Total *N* = 170	
	Mean	95 % CI	Mean	95 % CI	Mean	95 % CI
VIQ score	120.7	118.8–128.5	110.5	105.8–115.2	119.6	117.9–121.4
PIQ score	122.5	120.4–124.5	140.7	136.6–145.1	124.3	122.2–126.4
FIQ score	123.8	121.9–125.7	127.6	123.1–132.2	124.2	122.4–125.9

**Fig. 3:**
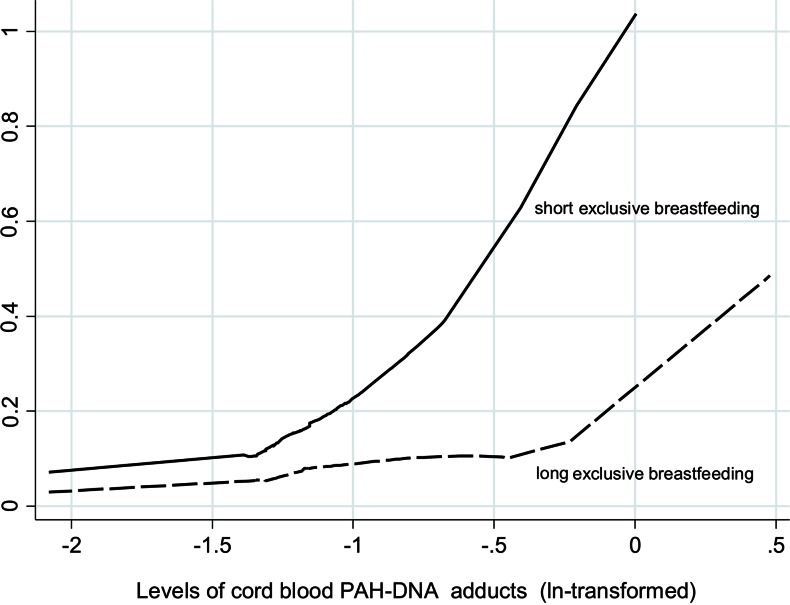
Predicted probability of DepVIQ (estimated from multivariable binary regression) by the levels of cord blood adducts (ln-transformed) and the breastfeeding practice (cutoff 6 months)

In order to assess the impact of prenatal PAH exposure on the depressed verbal IQ of children, binary multivariable regression analysis was performed including a set of potential confounding variables (Table 4). ETS variable was not included in the model due to its potential colinearity with PAH exposure. Of all independent variables considered in the model, only the level of cord blood PAH–DNA adducts (ln-transformed) and postnatal indoor PAH level had significant negative impact on the cognitive dysfunction of children. While the relative risk (RR) estimate for DepVIQ increased threefold with one ln-unit of cord blood adducts (RR = 3.0, 95 % CI 1.3–6.8), postnatal indoor airborne PAH levels had a weaker impact (RR = 1.6, 95 % CI 1.1–2.5). Neither child’s gender nor maternal education nor parity had a significant impact on the occurrence of DepVIQ. It is important to mention that the long-term exclusive breastfeeding (at least 6 months) significantly decreased the risk by about 70 % (RR = 0.3, 95 % CI 0.1–0.8) (Fig. 3). Interaction term for the birth season and cord blood adducts was insignificant (RR = 1.4; 0.2–10.1).

**Table 4:** Summary of the binary regression analysis for variables predicting RRs for depressed verbal IQ score in 7-year-olds

Predictors	Risk ratio	*z*	*p* > *z*	95 % confidence interval	
Cord blood adducts (ln-transformed)	3. 00	2.63	0.009	1.32	6.79
Birth season^a^	0.36	−0.84	0.403	0.03	3.99
Interaction term (birth season × cord blood adducts)	1.43	0.36	0.717	0.20	10.05
Indoor airborne PAH exposure (ln-transformed)	1.63	2.21	0.027	1.06	2.53
Maternal education (years of schooling)	1.06	0.66	0.510	0.89	1.25
Gender of child (girls)	0.89	−0.26	0.795	0.37	2.15
Parity^b^	1.80	1.27	0.205	0.73	4.44
Exclusive breastfeeding (6 months or longer)	0.31	−2.18	0.029	0.11	0.89

^a^Birth season: summer season 0 and winter season 1

^b^Parity: first childbearing 0 and two or more childbearings 1

## Discussion

To our knowledge, this is the first epidemiologic study showing that prenatal PAH exposure measured by cord blood PAH–DNA adducts is associated with cognitive dysfunction (DepVIQ) assessed by the WISC-R test, which is the commonly used instrument for measuring intelligence of children. The estimated relative risk of DepVIQ increased threefold with one ln-unit of prenatal PAH exposure (RR = 3.0; 95 % CI 1.3–6.8), and the effect of postnatal PAH exposure appeared to have a weaker impact (RR = 1.6; 95 % CI 1.1–2.5). The association between the cord blood PAH–DNA adducts and depressed verbal IQ score was not attenuated in the multivariable regression model after accounting for potential confounders.

PAH compounds have previously been shown to be a neurodevelopmental toxicant in both experimental and epidemiological studies (Calderon-Garciduenas et al. 2002; Chen and Schwartz 2009; Takeda et al. 2004), but the exact mechanism by which PAHs affect the developing brain remains unclear. Some studies have suggested the role of endocrine disruption (Archibong et al. 2002; Bui et al. 1986), binding to receptors for placental growth factors resulting in decreased exchange of oxygen and nutrients (Dejmek et al. 2000), binding to the human Ah receptor to induce P450 enzymes (Manchester et al. 1987), DNA damage resulting in activation of apoptotic pathways (Meyn 1995; Nicol et al. 1995; Wood and Youle 1995), or oxidative stress due to inhibition of the brain antioxidant scavenging system (Saunders et al. 2006). Recent studies suggest that prenatal exposures may affect epigenetic programming and immune, metabolic, and neurological functions, which may be manifested later in life (Barker 2004; Wilson and Jones 1983).

The strong effect of prenatal PAH exposure is consistent with human and experimental studies showing that the fetus and infant are more sensitive than adults to diverse environmental toxicants (Anderson et al. 2000; Grandjean and Landrigan 2006; National Research Council 1993; Perera et al. 2004; WHO 1986). Our results are also in agreement with other epidemiologic studies which have demonstrated an adverse effect of prenatal exposure to ambient air pollution on children’s cognitive development in cohorts in New York City (NYC), China, and Poland (Perera et al. 2006). For example, the Bayley Scales of Infant Development-Revised was used to assess children’s mental and psychomotor development at age 3 in a NYC cohort study. Children in the upper quartile of prenatal PAH exposure scored 5.7 points lower on the mental development index at age 3 than those in the lowest quartile of exposure to PAH (Perera et al. 2006). Also, Tang et al. (2008) evaluated the associations between prenatal ambient PAH exposure measured by PAH–DNA adducts, lead, and mercury on cognitive function measured by the Gesell Developmental Schedules at age 2 among children in China. After adjusting for potential confounders, increased PAH–DNA adduct levels were associated with decreased motor development quotients. The odds ratio of motor developmental delay was 1.91 (95 % CI 1.22–2.97) per 0.1-unit increase in PAH–DNA adducts. In the Polish cohort being earlier studied in Krakow, the Raven Colored Progressive Matrices, a nonverbal test of reasoning ability, was used to evaluate the effects on child intelligence at age 5 of prenatal PAH exposure estimated by personal air monitoring during pregnancy (Edwards et al. 2010). The effects were adjusted for potential confounders including socio-demographic factors and prenatal exposure to ETS. High prenatal exposure to PAH was associated with decreased child IQ at age 5. Similarly, follow-up of the NYC cohort through age 6 using the Wechsler Preschool and Primary Scale of Intelligence (WPPSI) showed a significant adverse effect on prenatal PAH exposure on child IQ at age 5 (Perera et al. 2009). Wang et al. (2009) examined the health effects of traffic-related air pollution on neurobehavioral functions among third-grade children. After adjusting for the children’s demographic, early childhood factors, and indoor air pollution, traffic-related ambient air pollution exposure was significantly associated with poorer performance on the visual simple reaction time-preferred hand (odds ratio (OR) = 1.67, *p* = 0.044), digit symbol (OR = 1.38, *p* = 0.019), and sign register (OR = 1.94, *p* = 0.001).

Our study confirmed a protective effect of longer exclusive breastfeeding on the cognitive function of children, which is explained by the fact that breastfed infants could have been influenced by omega-3-polusaturated fatty acids that are normally present in breast milk or other bioactive components essential for cognitive development (Crawford 1993; Lundqvist-Persson et al. 2010; Neuringer and Connor 1986). However, there are other possible mechanisms that may explain the association between breastfeeding and cognitive function since breastfeeding may be an indicator of a safe and sound maternal attachment status, which may have a positive influence on the child’s psychological development into later age. Breastfeeding may also be a marker of other unmeasured maternal characteristics such as maternal intelligence. In our analysis, we did not consider maternal intelligence as it was found that maternal education correlated significantly with maternal cognitive capacity.

The main limitation of the present study is the relatively small size of the sample of children who have undergone the cognitive evaluation. On the other hand, the analysis accounted for factors that are known to affect intellectual development, such as maternal tobacco smoke, exclusive breastfeeding, parity, or maternal education. A strength of the study also results from the fact that we assessed the intelligence of the children at the age of 7 when the IQ can be measured reliably. An exceptional advantage is that an individual prenatal exposure to PAH was based on cord blood PAH–DNA adducts, which integrate various sources of PAH exposure in prenatal period.

In conclusion, given the reported association between DepVIQ and learning disabilities, the findings suggest the need to reduce exposure of pregnant women to air pollution and other environmental sources of PAH.
